# Pulse Doppler ultrasound as a tool for the diagnosis of chronic testicular dysfunction in stallions

**DOI:** 10.1371/journal.pone.0175878

**Published:** 2017-05-30

**Authors:** Jose M. Ortiz-Rodriguez, Luis Anel-Lopez, Patricia Martín-Muñoz, Mercedes Álvarez, Gemma Gaitskell-Phillips, Luis Anel, Pedro Rodríguez-Medina, Fernando J. Peña, Cristina Ortega Ferrusola

**Affiliations:** 1 Laboratory of Equine Reproduction and Equine Spermatology, Veterinary Teaching Hospital, University of Extremadura, Cáceres, Spain; 2 Department of Animal Medicine, Surgery and Veterinary Anatomy, University of León, León, Spain; 3 Department of Zootechnical Sciences, University of Extremadura, Cáceres, Spain; Faculty of Animal Sciences and Food Engineering, University of São Paulo, BRAZIL

## Abstract

Testicular function is particularly susceptible to vascular insult, resulting in a negative impact on sperm production and quality of the ejaculate. A prompt diagnosis of testicular dysfunction enables implementation of appropriate treatment, hence improving fertility forecasts for stallions. The present research aims to: (1) assess if Doppler ultrasonography is a good tool to diagnose stallions with testicular dysfunction; (2) to study the relationship between Doppler parameters of the testicular artery and those of sperm quality assessed by flow cytometry and (3) to establish cut off values to differentiate fertile stallions from those with pathologies causing testicular dysfunction. A total of 10 stallions (n: 7 healthy stallions and n: 3 sub-fertile stallions) were used in this study. Two ejaculates per stallion were collected and preserved at 5°C in a commercial extender. The semen was evaluated at T0, T24 and T48h by flow cytometry. Integrity and viability of sperm (YoPro^®^-1/EthD-1), mitochondrial activity (MitoTracker^®^ Deep Red FM) and the DNA fragmentation index (Sperm Chromatin Structure Assay) were assessed. Doppler parameters were measured at three different locations on the testicular artery (Supratesticular artery (SA); Capsular artery (CA) and Intratesticular artery (IA)). The Doppler parameters calculated were: Resistive Index (RI), Pulsatility Index (PI), Peak Systolic Velocity (PSV), End Diastolic Velocity (EDV), Time Average Maximum Velocity (TAMV), Total Arterial Blood Flow (TABF) and TABF rate. The capsular artery was the most reliable location to carry out spectral Doppler assessment, since blood flow parameters of this artery were most closely correlated with sperm quality parameters. Significant differences in all the Doppler parameters studied were observed between fertile and subfertile stallions (p ≤ 0.05). The principal components analysis assay determined that fertile stallions are characterized by high EDV, TAMV, TABF and TABF rate values (high vascular perfusion). In contrast, subfertile stallions tend to present high values of PI and RI (high vascular resistance). The ROC curves revealed that the best Doppler parameters to predict sperm quality in stallions were: Doppler velocities (PSV, EDV and TAMV), the diameter of the capsular artery and TABF parameters (tissue perfusion parameters). Cut off values were established using a Youden´s Index to identify fertile stallions from stallions with testicular dysfunction. Spectral Doppler ultrasound is a good predictive tool for sperm quality since correlations were determined among Doppler parameters and markers of sperm quality. Doppler ultrasonography could be a valuable diagnostic tool for use by clinical practitioners for the diagnosis of stallions with testicular dysfunction and could be a viable alternative to invasive procedures traditionally used for diagnosis of sub-fertility disorders.

## Introduction

Testicular dysfunction in stallions is an important part of reproductive clinical medicine, and has a significant impact on the equine breeding industry. A reduction in ejaculate quality or sperm production can be triggered by acute processes such as testicular trauma, an increase in scrotal temperature, testicular torsion or due to inguinal hernias [1–5]. In these cases, if the cause is identified and treated early, semen quality may gradually return to normal values over time. However, in severe cases or idiopathic processes, the functionality of the testis can be severely affected and stallions can experience degenerative changes [6].

According to The Society of Theriogenology, the recommended protocol for assessing stallion fertility should include an ultrasound examination of the reproductive tract as well as an evaluation of sperm motility, morphology and sperm numbers [7]. The testicular volume and the Daily Sperm Output (DSO) are calculated using ultrasonography. The actual DSO is the number of sperm that one stallion can produce on a daily basis. In fact, one of the most widely used diagnostic criteria by clinicians to predict fertility problems is spermatic efficiency (actual DSO/predicted DSO). The main problem is that when changes in testicular size are detected, damage to the testis is already significant. In addition to this, a low spermatic efficiency coupled with a high percentage of abnormal sperm (including immature spermatogenic cells) in the ejaculate, have been used as diagnostic criteria for possible testicular degeneration [6].

The functionality of the testis is highly dependent on proper testicular perfusion. In fact, vascular disturbances are one of the most common causes of subfertility [2, 8]. Doppler ultrasound has become an indispensable tool for the clinical assessment of male fertility in andrology [9–11]. This technique is a good early indicator of acute pathologies related to vascular disorders, and is also a good predictor of semen quality in other species such as the dog and human [9, 12, 13]. However, despite considerable effort to validate this imaging modality in stallions, reference values have not yet been established [2, 14]. In addition, few studies have been undertaken to understand the changes to vascular perfusion in stallions with chronic subfertility. Furthermore, early diagnosis of testicular dysfunction triggered by vascular disturbance is crucial for the application of therapeutic strategies to maximize fertility and delay tissue damage in stallions. In addition, Doppler ultrasound is an excellent tool to monitor therapeutic outcome after medical or surgical treatments [4, 15].

Recently, new techniques in flow cytometry are being introduced to assess equine semen quality [16, 17]. These assays provide more specific information about sperm quality and function. Moreover, some of these tests such as the Sperm Chromatin Structure Assay (SCSA) show a close correlation with fertility in stallions and allow classification of the ejaculates based on fertility [18]. Flow cytometry also offers a major insight into molecular damage experienced by spermatozoa after processing, and has allowed the development of new approaches to improve fertility such as a colloidal centrifugation, formulation of new extenders and other strategies [19–22]. However, the elevated cost of flow cytometers and the few laboratories with available equipment are the main reasons explaining why these techniques are not commonly used yet in the equine breeding soundness diagnosis and work up. Furthermore, the application of other more economical and non-invasive diagnostic tools such as Doppler ultrasound could be a valuable alternative for practitioners. Assessing the vascular perfusion of the testicular artery could be a good early indicator of sperm dysfunction. For this reason, the aims of this research were: (1) to assess if Doppler ultrasonography is a good tool to diagnose stallions with testicular dysfunction; (2) to study the relationship between Doppler parameters of the testicular artery and those of sperm quality assessed using flow cytometry (membrane integrity, mitochondrial activity and SCSA (Sperm Chromatin Structure Assay), (3) to establish if there are differences in the blood flow between stallions with normal sperm function and stallions with testicular dysfunction (chronic processes); (4) to measure Doppler parameters of intra-testicular arteries in stallions to determine if they can be used as a predictor of sperm production or ejaculate quality; and, finally, (5) to define cut off values to differentiate fertile stallions from stallions with testicular dysfunction.

## Materials and methods

### Experimental design

This study aims to investigate whether the evaluation of testicular perfusion could be a good indicator of sperm dysfunction. For this purpose seven proven fertile stallions and three stallions with chronic subfertility problems (low sperm production and poor semen quality) were used. A basic spermiogram and a B-mode ultrasonographic examination were carried out on all stallions. Two ejaculates were evaluated per stallion to determine sperm quality using flow cytometry. Ejaculates were maintained refrigerated at 5°C for 48h. Samples were taken at the beginning of the incubation period and after, 24 and 48 hours to evaluate: Membrane integrity and viability, mitochondrial membrane potential and DNA fragmentation (SCSA).

All experiments were reviewed and approved by the Ethical committee of the University of Extremadura, Spain, (Ref AGL2013-43211-R).

### Reagents and media

Hoechst 33342 [(Ex: 350 nm, Em: 461 nm), (Ref: H3570)], ethidium homodimer (Eth) [(Ex: 528 nm, Em: 617 nm), (Ref: E1169)], YO-PRO-1 [(Ex: 491 nm; Em: 509 nm) (Ref: Y3603)], MitoTracker^®^ Deep Red ^FM^ [Ex: 644 nm, Em 665 nm) (Ref: 22426)] were purchased from Thermo Fisher Scientific (Molecular Probes). Acridine Orange hemi (zinc chloride) salt [(Ex: 490 nm, Em: 525 nm double stranded DNA and Emission 620 nm in presence of single chain fragmentation of the DNA) (Ref: A6010)] and Triton^™^ X-100 (Ref: 234729) were purchased from Sigma-Aldrich.

### Animals

Ten adult stallions of different breeds and ages (ranging from 6 to 18 years old) were used in this study. The stallions were kept at the Veterinary Teaching Hospital of the University of Extremadura. All stallions were handled and maintained according to the established institutional and European regulations (Law 6/2913 June 11th and European Directive 2010/63/EU). Seven out of ten of these horses were healthy stallions with proven fertility and without any previous history of reproductive disorders. The other three horses were referred to the Veterinary Teaching Hospital during the breeding season for different fertility problems, but with similar history of chronic subfertility. Stallion number #1 had been subjected to a unilateral castration three years before for a recurrent inguinal hernia, and presented poor semen quality; the second stallion (#2) had a history of subfertility with bilateral atrophy of both testes and had not been able to establish any successful pregnancy for the last 5 years. Finally, the last stallion (#3) presented an immune-mediated testicular vasculitis in both testes. During the breeding soundness evaluation, all three stallions had small soft testes, poor semen quality and a low sperm production (DSO).

### Ultrasonographic assessment

The ultrasound equipment used in this study was a MyLab30 VET^®^ (Esaote, Genova, Italy) with three different probes: 5–7.5 MHz linear transducer (LV513 VET^®^), 10–13 MHz linear transducer (LA523 VET^®^) and 3–9 MHz semi-convex transducer (CA123 VET^®^).

Prior to the ultrasound examination, a physical examination and a reproductive exploration of the reproductive tract was performed [23]. All ultrasound examinations were carried out by the same operator to avoid variation. The stallions were restrained in stocks and sedated with xylazine (0.5 mg/kg i.v) (Xilasyn^®^ 2, Virbac) IV [14].

#### B-Mode ultrasonographic evaluation

This imaging modality was used to identify the various anatomical structures of the testis and diagnose possible pathologies. The testicular volume (TV: 0.053 x height x length x width) and the estimated Daily Sperm Output (DSOe: [0.024 x TTV]– 0.76) were calculated, where TTV (Total Testicular Volume) is the sum of the volume of the left and the right testicle [24].

#### Colour and Pulse Doppler ultrasonographic evaluation

**Fig 1:**
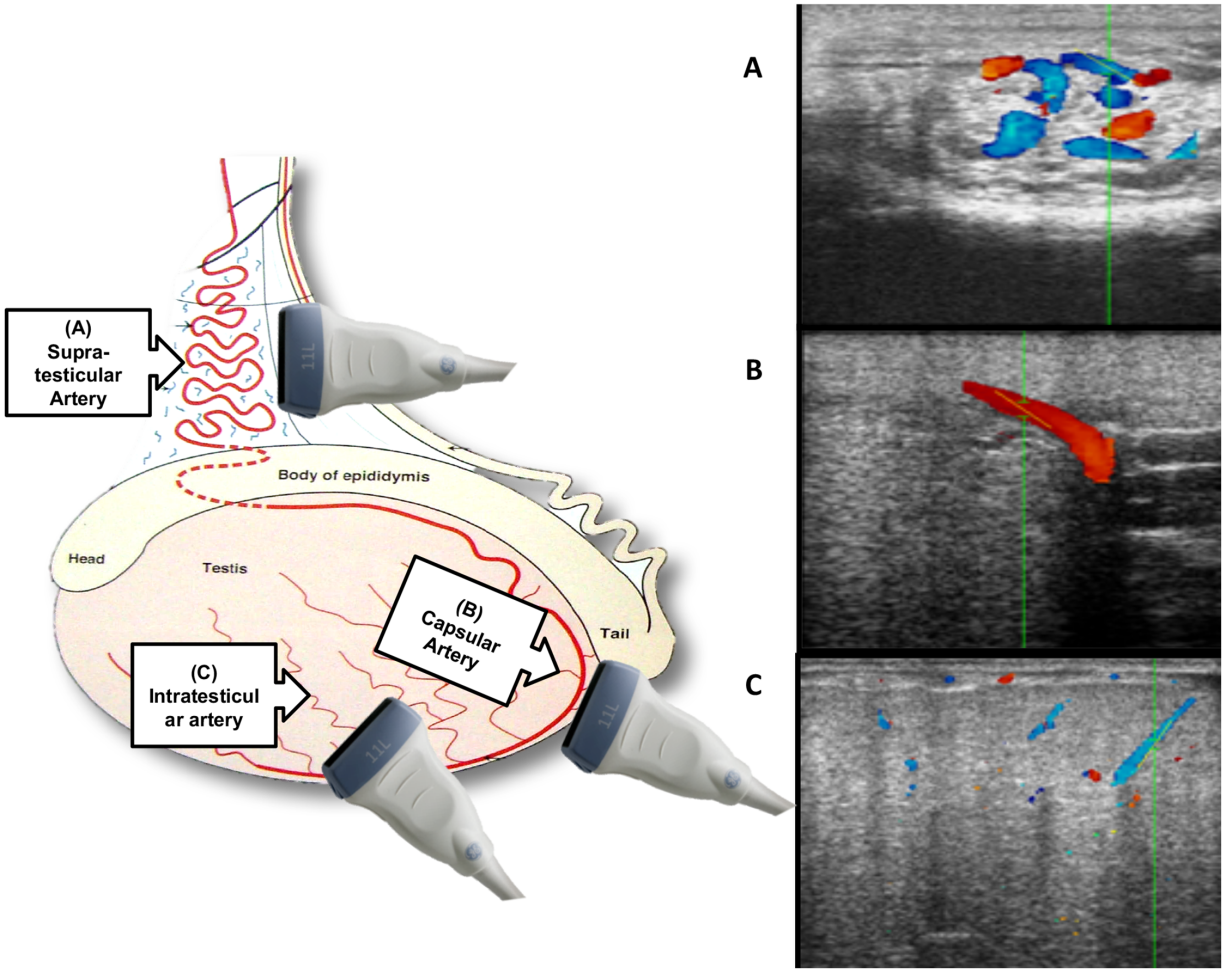
Testicular artery in three different locations and transducer orientation. A. Spermatic cord: Supratesticular artery (SA); B. Close to the tail of epididymis: Capsular artery (CA) and, C. Within the parenchyma: Intratesticular artery (IA). Modified image from the book “Ultrasonic imaging and animal reproduction: Color-Doppler ultrasonography,” O.J. Ginther.

The testicular artery was visualized in three locations: (1) in the spermatic cord (Supra-testicular artery (SA)); (2) at the epididymal edge of the testicle, close to the tail of the epididymis (Capsular artery (CA)) and (3) within the parenchyma, in the caudo-ventral two thirds of the testis (Intratesticular artery (IA)) (Fig 1).

**Fig 2:**
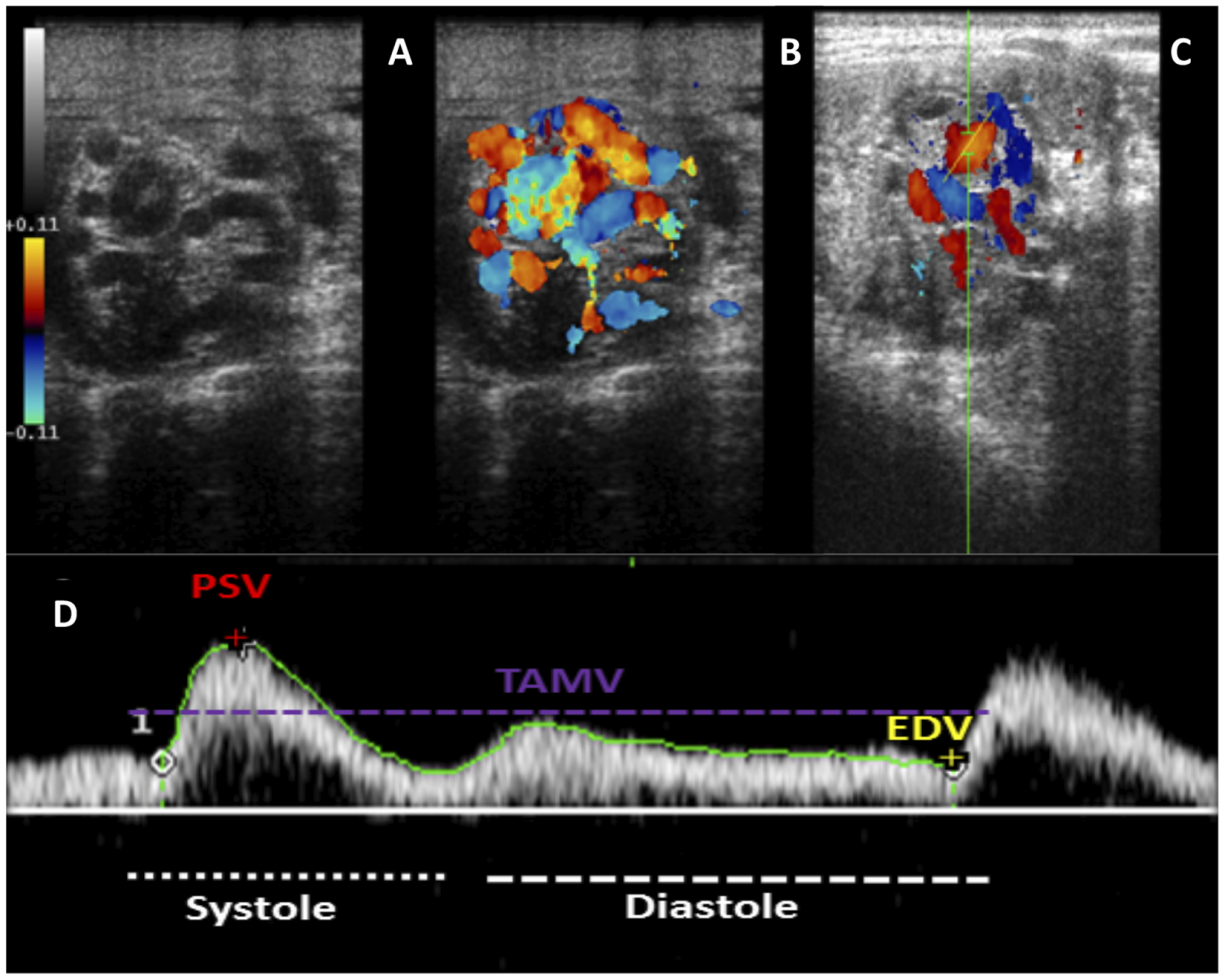
Cross section of the spermatic cord with the three different ultrasound modalities. A. B-Mode ultrasound (grey scale); B. Colour Doppler ultrasound of the spermatic cord´s vessels; C. Pulse Doppler ultrasound of the supratesticular artery within the spermatic cord. D. Display of the equipment used to measure a cardiac cycle using pulse Doppler. Three Doppler velocities calculated by the ultrasound equipment’s algorithm package.

Firstly, the B-mode and colour Doppler modality were performed to identify the arterial vessel (Fig 2A and 2B). Subsequently, Pulse Doppler was applied to quantify the velocity of the blood flow within the vessel (Fig 2C and 2D). Measurements were obtained according to previous studies [5, 14]. The angle of insonation used was between 30° and 60° [14, 25]. The ultrasound equipment’s algorithm package was used to calculate the velocities and Doppler indices (Fig 2D). A single mean value from 3 identical waveforms for each measurement at each location of the artery was obtained.

The Doppler parameters calculated were: The Peak Systolic Velocity (PSV), the End Diastolic Velocity (EDV) and the Time Average Maximum Velocity (TAMV), Resistive Index (RI: PSV-EDV/PSV) and Pulsatility Index (PI: PSV-EDV/TAMV). Total arterial blood flow (TABF: TAMV x A; where A: *πr*^2^ is the cross sectional area of the CA), and total arterial blood flow rate (TABF rate: TABF/TTV x 100) were also calculated for all stallions as indicators of testicular perfusion [26] (Table 1).

**Table 1:** Doppler parameters assessed in this study.

**Doppler Velocities**	
**PSV**	Peak Systolic Velocity
**EDV**	End Diastolic Velocity
**TAMV**	Time Average Medium Velocity
**Doppler Indices**	
**PI** (Pulsatility Index)	PSV-EDV/TAMV
**RI** (Resistive Index)	(PSV- EDV)/PSV
**Tissue Perfusion Parameters**	
**TABF** (Total Arterial Blood Flow)	TAMV x A; A: *πr*^2^
**TABF rate** (Total Arterial Blood Flow rate)	TABF/TTV x 100

### Semen collection and processing

A mean of 7–10 collections per stallion (one collection/day) were performed to empty extragonadal sperm reserves before starting the experiment. In this study a total of two ejaculates per stallion were used to assess the quality of semen by means of flow cytometry [23, 24].

The stallion’s penises were cleansed with warm water and thoroughly dried to avoid contamination of the samples. All the ejaculates were collected using a pre-warmed (45–50°C) Missouri model artificial vagina, filled with non-spermicidal lubricant, to which an inline nylon micromesh filter was attached to separate both debris and the gel fraction. The semen was immediately transported to the laboratory for evaluation and processing. In the laboratory, the gel fraction was removed from the ejaculate and the volume of the gel free fraction of the ejaculate was measured in a test tube. Sperm concentration was determined using a spectrophotometer (Spermacue^®^, Minitüb Ibérica, La Selva del Camp, Spain). Afterwards, the actual DSO (DSOa: Volume x Concentration) was calculated and spermatic efficiency ((DSOa/DSOe) x 100) of each stallion was estimated [7].

The semen samples (two per stallion) were extended 1:1 (v:v) in INRA 96^®^ (IMV, Aigle, France); centrifuged (600g for 10 min) and re-suspended again in the same commercial extender to a final concentration of 50 x 10^6^ sperm/ml. The samples were kept at 5°C for 48h. Samples were analysed initially (T0), after 24 hours (T24) and after 48 hours (T48) to evaluate semen quality.

### Sperm motility analysis

The motility and kinematic parameters of the sperm were assessed using a CASA system (ISAS^®^ Proiser Valencia Spain) [27]. Semen was extended in INRA 96 to a final concentration of 50 x 10^6^ spz/ml and was loaded into a 20μm deep Leja chamber (Leja Amsterdam, the Netherlands). The analysis was based on the examination of 60 consecutive, digitalized images obtained from three random fields, using a x10-negative phase contrast objective and a warmed stage (37°C). Images were taken with a time lapse of 1 s. The number of particles incorrectly identified as spermatozoa were minimized on the monitor by using the playback function. With respect to the parameter settings for the program, spermatozoa with a VAP < 15 μm/s were considered immotile, while spermatozoa with a velocity >15 μm/s were considered motile. Spermatozoa deviating < 45% from a straight line were designated linearly motile and spermatozoa with a circular velocity (VCL) > 45 μm/s were designated rapid sperm. Sperm motion and calculated kinematic parameters measured by CASA included: Curvilinear Velocity (VCL) μm/s; Linear Velocity (VSL) μm/s; Mean Velocity (VAP) μm/s [27].

### Flow cytometry assessment

Multiparametric flow cytometry analysis was conducted using a MACSQuant^®^ Analyser 10 (Miltenyi Biotech) flow cytometer equipped with three lasers emitting at wavelengths of 405 nm, 488 nm, and 635 nm and 10 photomultiplier tubes (PMTs) (V1 (excitation 405 nm, emission 450/50 nm), V2 (excitation 405 nm, emission 525/50 nm), B1 (excitation 488 nm, emission 525/50 nm), B2 (excitation 488 nm, emission 585/40 nm), B3 (excitation 488 nm, emission 655–730 nm (655LP + split 730), B4 (excitation 499 nm, emission 750 LP), R1 (excitation 635 nm, emission 655–730 nm (655LP + split 730) and R2 (excitation 635 nm, emission filter 750 LP). The system was controlled using MACSQuantify^®^ software. The equipment was calibrated daily with calibration beads provided by the manufacturer and compensation overlap performed before each particular experiment.

Flow cytometric analysis of SCSA was performed with a Coulter EPICS XL (Coulter Corporation Inc.) at 15mW, at 488 nm, analysed by the EXPO 2000 software. Forward and sideways light scatter were recorded for a total of 10000 events per sample, and flow rate was maintained at 200–300 cells/s. Green fluorescence was detected in FL1, while orange fluorescence was detected in FL2 and red fluorescence in FL3. For the SCSA, both FL1 and FL3 photodetectors were used.

#### Assay for sperm viability, membrane integrity and active mitochondria

A combination of Hoechst 33342, Yo-Pro-1 and ethidium homodimer (Eth) was used to study viability of sperm and membrane integrity [28] and Mitotracker Deep Red was used to stain active mitochondria [29].

**Fig 3:**
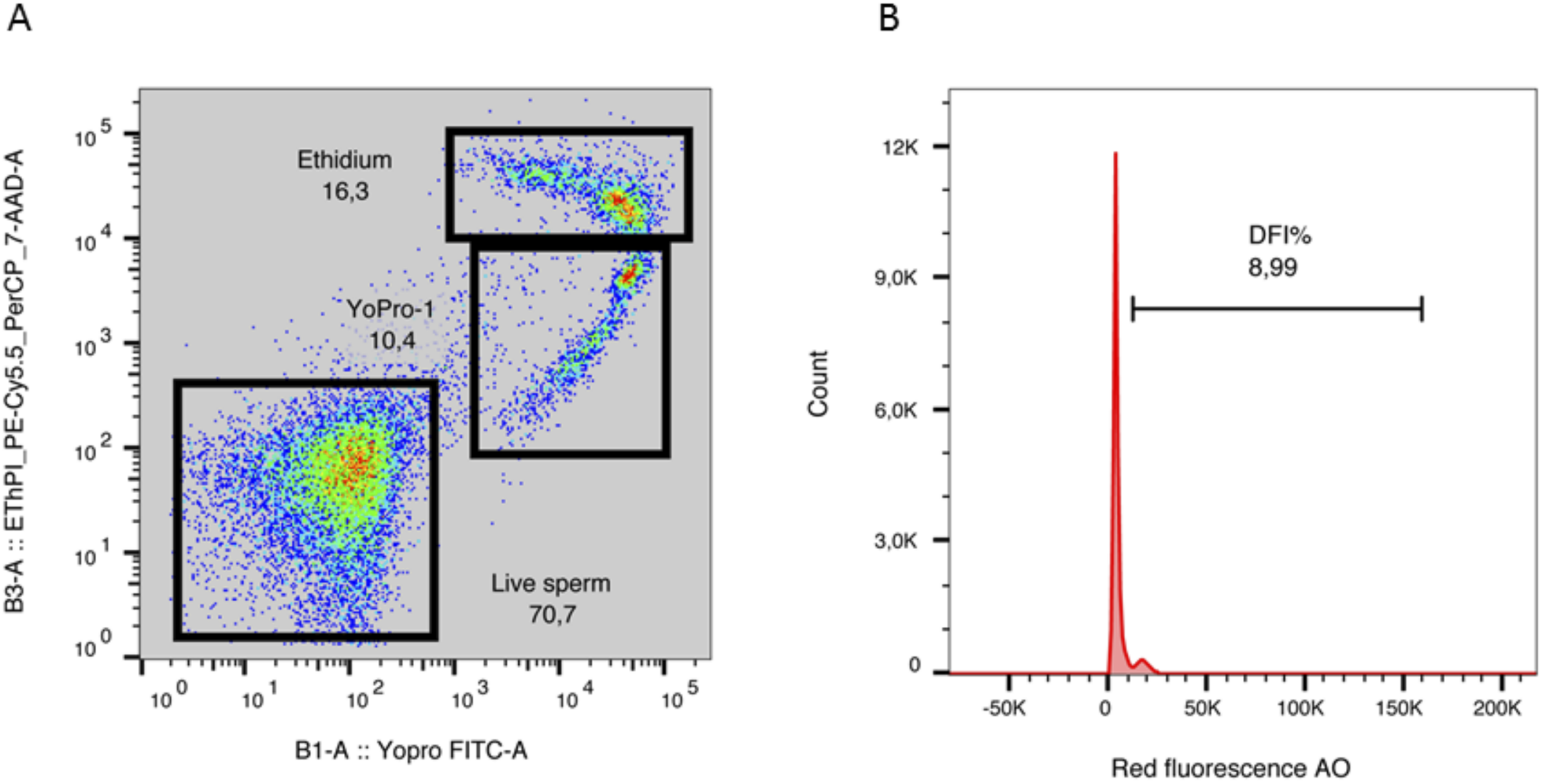
Flow cytometry detection of sperm viability and membrane integrity (A) and DNA fragmentation Index (DFI) (B) of stallion sperm. (A) Representative density plot graphic with the three subpopulations of sperm: Live sperm (unstained spermatozoa), the Yo-Pro-1 positive cells (sperm in the early stages of apoptosis) and spermatozoa stained with Ethidium (dead sperm). (B) Representative histograms of DFI (%) (Sperm Chromatin Structure assay).

In brief, 5 x 10^6^ spermatozoa were extended in a final volume of 1 ml of Phosphate Buffered Saline solution (PBS). This suspension was stained with 0.3 μL of Hoechst 33342 (22.5 μM), 1 μL of Yo-Pro-1 (25 μM) and 0.1 μL of Mitotracker Deep Red (500 μM). After thorough mixing, the sperm suspension was incubated at room temperature in the dark for 25 min. Then, 0.3 μL of Eth (1.167 mM) was added and the mixture was incubated for 5 minutes at room temperature and analysed. Forward and sideways light scatter were recorded for a total of 50,000 events per sample. Non-sperm events were eliminated by gating the sperm population after Hoechst 33342 staining. The results of sperm viability and membrane integrity were visualised in a density plot graphic. This distinguishes three sperm subpopulations. The first one is the subpopulation of unstained spermatozoa. These spermatozoa are considered alive with no membrane alteration. The second one are the Yo-Pro-1 positive cells emitting green fluorescence. This subpopulation of sperm are in the early stages of apoptosis [30]. Finally, the last subpopulations of dead spermatozoa were easily detected: apoptotic spermatozoa stained with Ethidium homodimer (emitting red fluorescence) (Fig 3A; S2 Dataset and S1 File).

Multiparametric flow cytometry allows simultaneous evaluation of spermatic viability and mitochondrial activity in the same sample with the H33342, Ethidium homodimer and Mitotracker Deep Red probes, respectively. The Mitotracker Deep Red positive cells emitting deep red fluorescence, which corresponds with live spermatozoa with highly active mitochondria. Another population is composed of spermatozoa stained with both probes, emitting deep red and red fluorescence. Other population are necrotic spermatozoa with inactive mitochondria, stained only with Ethidium homodimer (emitting red fluorescence). This protocol is a modified version of previously published protocols by our research group [28, 29].

#### Sperm chromatin structure assay

The sperm chromatin structure assay (SCSA) is a method to determine the susceptibility of sperm DNA to undergo acid induced denaturalization in situ [31]. Following exposure of the prepared DNA to acridine orange (AO), the degree of chromatin integrity was analysed by flow cytometric measurement of the metachromatic shift from green (stable, double-stranded DNA) to red (denatured, single-stranded DNA) AO fluorescence. Each seminal sample was extended in TNE buffer (0.15 M NaCl, 0.01 M Tris-HCL, 1 mM EDTA (ethylenediaminetetra-acetic acid), pH 7.4) to obtain a final sperm concentration of 1–2 x 10^6^ spermatozoa/mL. TNE-extended spermatozoa (200 μL) were subjected to partial DNA denaturation *in situ* (by mixing with 400 μL of a low pH detergent solution containing 0.17% Triton X-100, 0.15 M NaCl and 0.08 N HCl, pH 1.2), followed 30 seconds later (incubation at room temperature) by staining with 1.2 mL of AO (6 μg/ml in 0.1 M citric acid, 0.2 M Na_2_HPO_4_, 1 mM EDTA, 0.15 M NaCl, pH 6.0). The stained samples were analysed within 3 minutes of AO staining by the flow cytometer. AO is characterized as depicting green fluorescence when it intercalates into native double-stranded DNA, and red fluorescence if DNA is single stranded. Green fluorescence was detected in the FL1 photodetector, while red fluorescence was detected in FL3. The amount of red and green fluorescence emitted was measured on a total of 10,000 spermatozoa per sample, and flow rate was maintained at 200–300 cells/s, allowing calculation of the DNA fragmentation index (%DFI). The percentage of DNA fragmentation index is given by the ratio of cells with single-stranded DNA (ss DNA) to total cells (ss DNA and ds DNA), reflecting the loss of sperm DNA integrity [32] (Fig 3B; S2 Dataset and S1 File).

### Statistical analysis

The data were first examined using the Kolmogorov- Smirnov and chi-squared tests to determine their distribution. A Levene´s test was used to assess the homogeneity of variances for the variables calculated. In view of the non-Gaussian distribution of the data gathered, a non-parametric Kruskal-Wallis test was used. Differences were considered significant when *p < 0*.*05*. The results are displayed as a mean ± SD.

A principal component analysis was used to reduce the number of Doppler variables able to identify fertile and subfertile stallions [33].

The correlations between Doppler parameters and seminal quality parameters were investigated using Spearman’s correlation test. Significant correlations were determined when *p < 0*.*05*.

Receiver operating characteristic (ROC) curves and Youden’s J statistics were used to investigate the value of the proposed variables as indicators of sperm quality and cut-off values were also established. Receiver operating characteristics (ROC) analyses were used expressing prognostic value as area under curve (AUC) with a 95% confidence interval (CI) and significance test [34]. Results were expressed as mean ± SEM.

All analyses were performed using SPSS version 21.0 for Windows.

## Results

### Sperm motility and kinematics

All parameters of sperm motility and kinematics were lower in sub-fertile than in fertile stallions (p ≤ 0.05) (Table 2).

**Table 2:** Sperm motility and kinematic parameters of fertile and subfertile stallions.

Stallions	TM *(%)*	PM *(%)*	VCL *(μm/sec)*	VSL *(μm/sec)*	VAP *(μm/sec)*
**Fertile**	89.38 ± 5.00^a^	67.38 ± 9.07^a^	109.10 ± 15.01^a^	60.19 ± 9.76 ^a^	85.43 ± 12.60^a^
**Sub-fertile**	51.67 ± 4.98^b^	33.67 ± 8.19^b^	104.33 ± 9.48^b^	44.17 ± 7.36^b^	69.33 ± 15.87^b^

Mean values and Standard Deviations (Mean ± SD) of TM (Total Motile), PM (Progressive Motile), VCL (Mean curvilinear velocity), VSL (Mean straight-line velocity), VAP (Average path velocity) in both groups (fertile and subfertile). Values with different superscripts differ (a. b;* p < 0*.*05)*.

### Ultrasonography assessment

#### B-Mode ultrasound

Hydrocele, varicocele and abnormalities in the echogenicity of the parenchyma were detected using ultrasound in sub-fertile stallions. Significant differences among fertile and sub-fertile stallions were also obtained for TTV, expected DSO, and actual DSO. The spermatic efficiency of sub fertile stallions was 54.69% vs. 96.3% in fertile stallions (Table 3).

**Table 3:** Measurements of Total Testicular Volume and DSOe with B-Mode ultrasonography and parameters of sperm production and testicular efficiency in fertile and subfertile groups.

Stallions	TTV (cm^3^)	DSO e (10^6^ spz)	DSO a (10^6^ spz)	Testicular efficiency (%)
***Fertile***	390 ± 71^a^	8110 ± 1710^a^	7810 ± 1648^a^	96.30^a^
***Sub-fertile***	155 ± 27^b^	4187 ± 2232^b^	2290 ± 788^b^	54.69^b^

Mean values (Mean) and Standard deviations (SD) of TTV (Total Testicular Volume), DSOe (expected Daily Sperm Output), DSOa (actual Daily Sperm Output), and Testicular efficiency ((DSOa/DSOe) x 100) in both groups (fertile and subfertile). Values with different superscripts differ *(a*, *b; p < 0*.*05)*.

#### Pulse Doppler

It was feasible to obtain all Doppler parameters at all three locations of the artery that were evaluated. The values of parameters decreased as the artery coursed from the spermatic cord to intratesticular locations.

Supratesticular artery: Doppler parameters tend to be higher in subfertile stallions vs. fertile stallions, although there were not any significant differences between groups at this location (Table 4; S1 Dataset and S1 File).

**Table 4:** Blood flow parameters of the supratesticular artery in fertile and subfertile stallions.

Supra-Testicular artery		
	Fertile	Subfertile
**PI**	2.28 ± 0.45	3.04 ± 1.22
**RI**	0.80 ± 0.05	0.80 ± 0.09
**PSV**	24.96 ± 6.58	26.91 ± 7.88
**EDV**	4.85 ± 1.34	5.12 ± 2.57
**TAMV**	8.82 ±1.89	8.13 ± 3.86

Mean values and Standard error of the mean (Mean ± SEM). PI (Pulsatility Index: PSV-EDV/TAMV); RI (Resistive index: PSV-EDV/PSV); PSV (Peak Systolic Velocity; EDV (the End Diastolic Velocity); and the TAMV (Time Average Maximum Velocity); Values with different superscripts differ (a. b;* p < 0*.*05)*.

**Fig 4:**
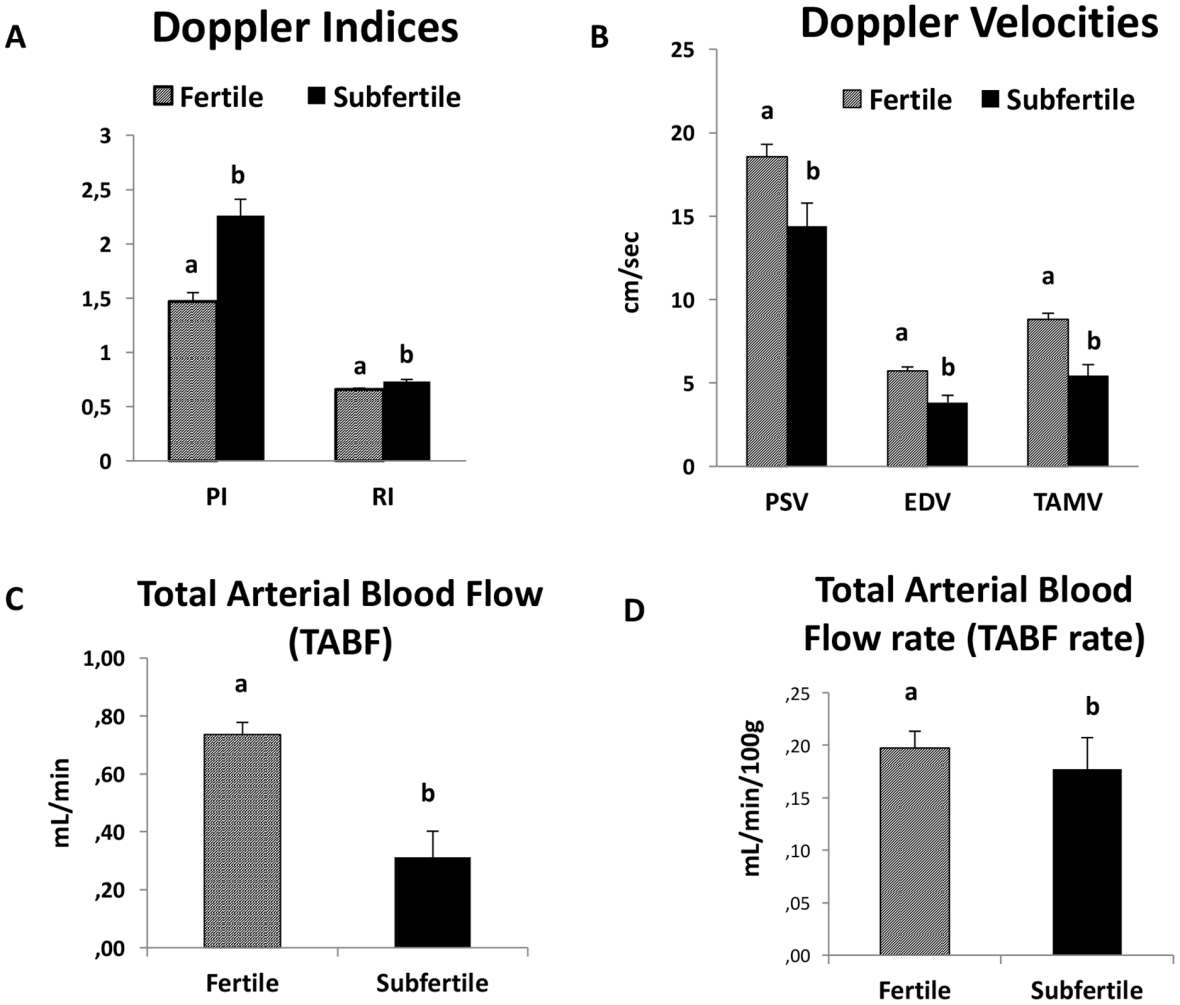
Mean values and standard error of the mean of Doppler parameters measured in the capsular artery in fertile and subfertile stallions. (A) Doppler Indices: PI (Pulsatility Index: PSV-EDV/TAMV) and RI (Resistive Index: PSV-EDV/PSV). (B) Doppler Velocities: PSV (Peak Systolic Velocity; EDV (the End Diastolic Velocity); and the TAMV (Time Average Maximum Velocity). (C) Total Arterial Blood Flow (TABF: TAMV x A; where A: *πr*^2^ is the area of the cross section of the CA). (D) Total Arterial Blood Flow rate (TABF rate: TABF/TTV x 100). TABF and TABF rate were calculated as indicators of testicular perfusion. Values with different superscripts differ (a. b;* p < 0*.*05)*. (S1 Dataset and S1 File).

Capsular artery: This artery was the easiest location for the practitioner to detect blood flow. Significant differences in all Doppler parameters were observed between fertile and subfertile stallions (p ≤ 0.05). The subfertile stallions had higher Doppler index values (lower perfusion) and lower velocities than fertile stallions (Fig 4; S1 Dataset and S1 File). Conversely, the total testicular perfusion assessed by TABF and TABF rate was lower in stallions with fertility problems.

Intratesticular artery: Doppler parameters were determined for the first time in intratesticular arteries. The position of these arteries and their small diameter caused measurement of Doppler parameters to be tedious and time-consuming. Once again, the PI and the RI were higher in subfertile stallions, although significant differences were not detected. EDV was significantly lower in horses with fertility problems (Table 5; S1 Dataset and S1 File)

**Table 5:** Blood flow parameters of the Intratesticular artery in fertile and subfertile stallions.

Intra-testicular artery		
	Fertile	Subfertile
**PI**	0.90 ± 0.21	0.97 ± 0.25
**RI**	0.57 ± 0.08	0.62 ± 0.09
**PSV**	10.08 ± 2.59	8.09 ± 0.81
**EDV**	4.26 ± 1.14^a^	3.40 ± 0.84^b^
**TAMV**	6.50 ± 1.64	5.81 ± 0.78

Mean values and Standard error of the mean (Mean ± SEM). PI (Pulsatility Index: PSV-EDV/TAMV); RI (Resistive index: PSV-EDV/PSV); PSV (Peak Systolic Velocity; EDV (the End Diastolic Velocity); and the TAMV (Time Average Maximum Velocity); Values with different superscripts differ (a. b;* p < 0*.*05)*. (S1 Dataset and S1 File).

**Fig 5:**
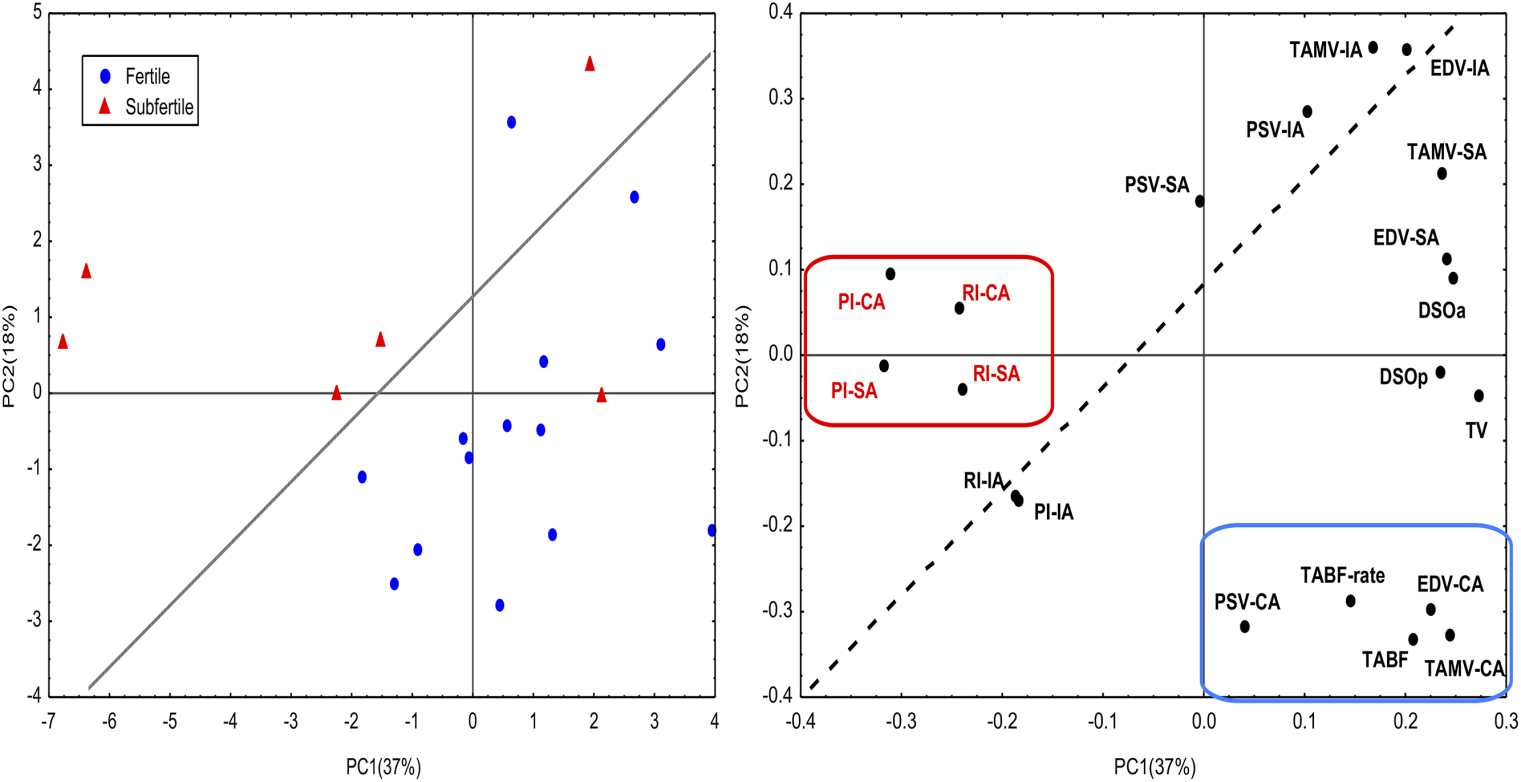
The principal component analysis assay (PCA) applied to Doppler parameters to identify fertile and subfertile stallions as described in materials and methods. Fertile stallions (1) are characterized by high values of TABF ratio, TABF, VDF and TAMV (right lower quadrant). Subfertile stallions (2) are categorized by high values of PI and RI in the supratesticular artery (TC: Testicular Cord) and in the capsular artery (PT) (left upper quadrant). (S1 File).

The principal component analysis assay determined that fertile stallions had high values for PSV, EDV, TAMV, TABF and TABF rate (high vascular perfusion). In contrast, subfertile stallions showed high values of Doppler indices in both locations (ST and CA) (Fig 5; S1 File))

### Assessment of the viability and integrity of the membrane

**Fig 6:**
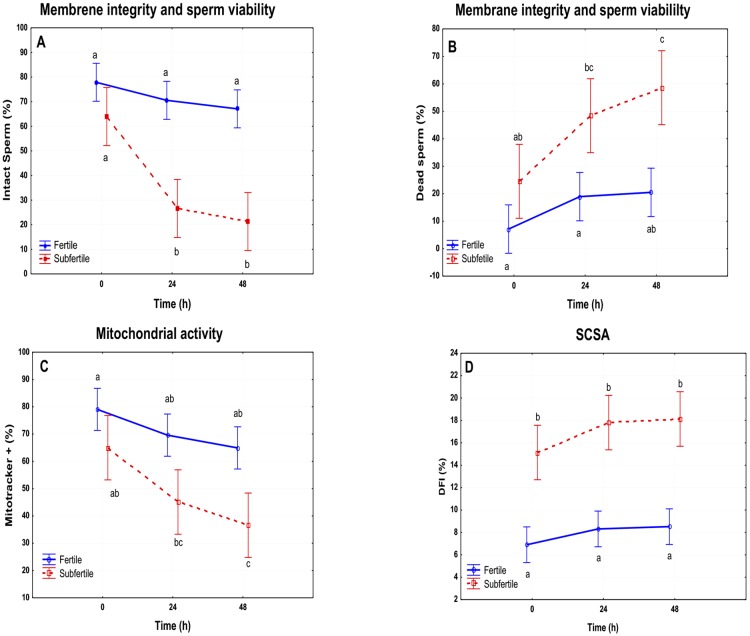
Graphical representation of the results obtained by flow cytometry in fertile vs subfertile stallions. A. Membrane integrity and sperm viability of sperm: Percentage of intact sperm at T0, T24 and T48 h of refrigeration. B Membrane integrity and sperm viability of sperm: Percentage of dead sperm at T0, T24 and T48 h of refrigeration. C. Mitochondrial activity: percentage of active mitochondria on sperm at T0, T24 and T48 h. D. DNA fragmentation Index of sperm at T0, T24 and T28h. a, b; *p ≤ 0*.*05*.

At T0 there were not any significant differences in the number of intact sperm between fertile and subfertile horses (77.87% vs. 63.95%). The percentage of intact sperm decreased concurrently with longer incubation times in both groups. However, only subfertile stallions underwent a drastic reduction in the percentage of intact sperm both at 24h (26.59%) and 48h (21.58%) *(p ≤ 0*.*05*). There were not significant changes in fertile stallions (Fig 6A; S2 Dataset and S1 File).

Similarly, there was an increase of dead sperm with time. Fertile stallions presented a significant change at 48h (p ≤ 0.05), but the percentage of dead sperm was lower than in subfertile stallions (T0: 7.14% vs 24.52%; T24: 18.93% vs 48.39%; T48: 20.49% vs 58.59%). Subfertile stallions presented at 48 hours 64.51% dead sperm (Fig 6B; S2 Dataset and S1 File).

### Mitochondrial activity

Fertile stallions presented higher percentages of active mitochondria and there were not any significant changes with time (Fig 6C). However, subfertile stallions showed lower mitochondrial activity than fertile ones (*p≤0*.*05*) and a significant decrease was noted after 48h of storage at 5°C (Fig 6C; S2 Dataset and S1 File).

### Fragmentation DNA Index (DFI)

Subfertile stallions showed higher values of DFI than fertile stallions (Table 6). However, there were not significant changes in DFI with time in either group (Fig 6D; S2 Dataset and S1 File).

**Table 6:** DNA fragmentation index (%DFI) of individual fertile (stallion1-7) and subfertile stallions (stallion 8–10) at T0, T24 and T48h of refrigeration.

	% DFI T0	% DFI T24	% DFI T48
	Mean ± SD	Mean ± SD	Mean ± SD
**Stallion 1**	5.27 ± 1.53	6.94 ± 1.08	7.33 ± 1.44
**Stallion 2**	4.97 ± 0.23	6.60 ± 1.00	6.57 ± 1.32
**Stallion 3**	7.12 ± 2.38	6.23 ± 0.64	7.81 ± 0.56
**Stallion 4**	6.44 ± 0.64	6.03 ± 1.43	5.29 ± 0.24
**Stallion 5**	7.80 ± 0.74	10.25 ± 2.65	8.53 ± 1.35
**Stallion 6**	9.08 ± 0.05	9.69 ± 0.06	9.65 ± 0.02
**Stallion 7**	7.61 ± 4.07	12.42 ± 2.01	14.42 ± 4.75
**Stallion 8**	14.54 ± 1.15	15.42 ± 2.13	18.32 ± 1.32
**Stallion 9**	16.73 ± 5.87	21.19 ± 2.66	22.94 ± 0.79
**Stallion 10**	15.14 ± 2.36	17.81 ± 0.27	18.13 ± 0.26

Values are shown following the model Mean ± Standard Deviation.

### Correlation between Doppler and flow cytometer parameters

Supratesticular artery: Doppler parameters/viability: there was a high correlation between Doppler indices and percentage of ethidium + at 24h (PI r: 0.733; RI r: 0.661. p≤0.05). A negative correlation was detected between EDV and dead sperm at 24 and 48h (r_T24_: -0.733; r_T48:_ - 0.661; p < 0.05) (Table 7).

**Table 7:** Correlations obtained by Spearman test of non-parametric values of Doppler parameters and those of flow cytometry at T0, T24 and T48h. *P < 0*.*05*.

**Capsular artery**	**PI**	**RI**	**PSV**	**EDV**	**TAMV**
Intact sperm T0				0.745	
Dead sperm T0				-0.721	
Dead sperm T24		0.705			
Intact sperm T48		-0.675			
Active mitochondria T0				0.709	
Active mitochondria T24	-0.673	-0.729			
Active mitochondria T48	-0.673	-0.729			
DFI (SCSA) T0				-0.709	
DFI (SCSA) T24				-0.733	
DFI (SCSA) T48		-0.723		-0.745	
**Supratesticular artery**	**PI**	**RI**	**PSV**	**EDV**	**TAMV**
Dead sperm T24	0.733	0.661		-0.733	
Dead sperm T48				-0.661	
**Intratesticular artery**	**PI**	**RI**	**PSV**	**EDV**	**TAMV**
DSO actual				0.685	0.721

Abbreviations: Pulsatility index (PI); Resistive index (RI) Peak systolic velocity (PSV); End diastolic velocity (EDV) and Time average maximum velocity (TAMV).

Capsular artery: Doppler parameters obtained from the capsular artery were most closely correlated with sperm quality parameters. Significant correlations between Doppler parameters and sperm quality parameters (Membrane integrity and viability. Mitochondrial activity and DNA fragmentation (DFI)) are represented in Table 7.

Intratesticular artery: TAMV and EDV showed high correlations with actual DSO (TAMV, r: 0.721; EDV, r: 0.685; *p≤ 0*.*05*) (Table 7).

### Prediction of the outcome of sperm quality using ROC curves

**Fig 7:**
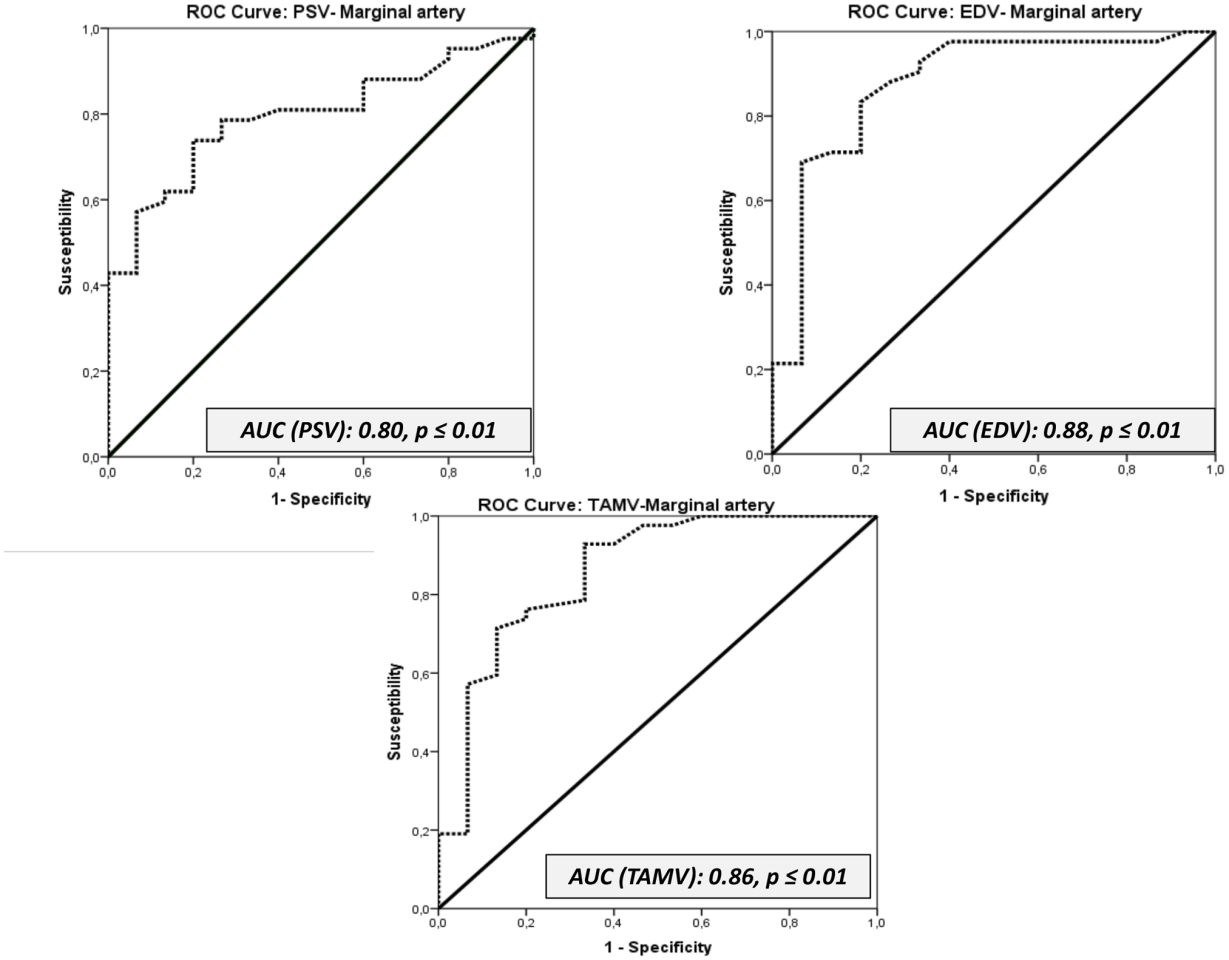
Receiver operating characteristic (ROC) curves for the parameters PSV, EDV, TAMV. AUC: Area under the Curve.

The Doppler parameters that were significantly correlated with sperm quality parameters were further investigated using ROC curves and Youden’s J index statistics. Several Doppler parameters in the capsular artery with potentially high predictive values of sperm quality were identified (Figs 7 y 8; S1 File).

**Fig 8:**
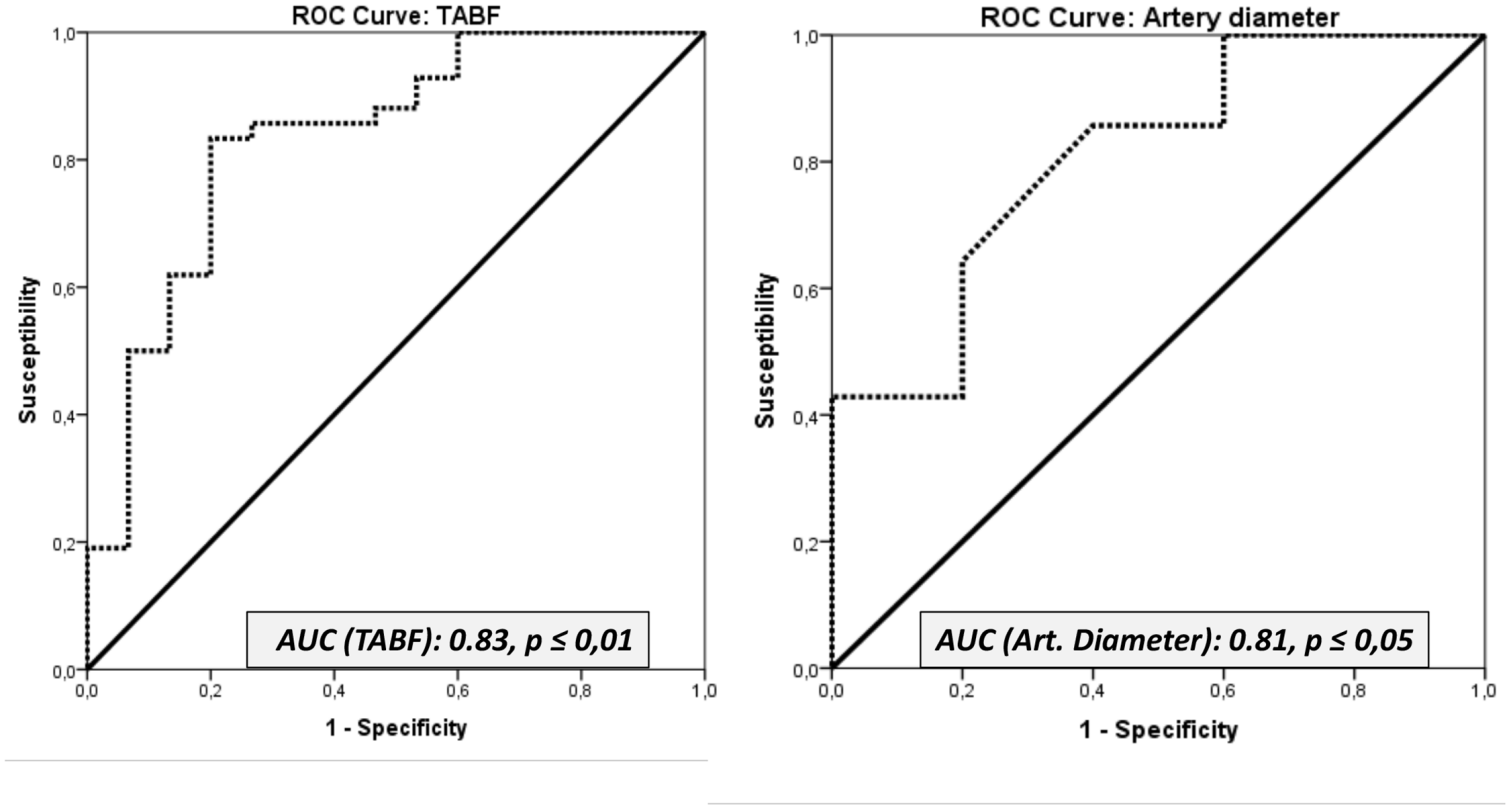
Receiver operating characteristic (ROC) curves for the parameters TABF and the diameter of the capsular artery. AUC: Area under the Curve.

The ROC curves revealed the better Doppler parameters to predict sperm quality of fertile stallions: PSV, EDV, TAMV, TABF, and the diameter of the capsular artery. The cut-off values of these parameters to differentiate fertile from subfertile stallions were established using Youden’s J statistics. The results are presented in the table (Table 8; S1 File).

**Table 8:** The area under the curve (AUC) of receiver operating characteristic (ROC) curves and Youden’s J statistics were applied to Doppler velocities of the capsular artery and TABF and artery diameters to investigate the value of the proposed variables as indicators of sperm quality.

Parameter	AUC	Cut off value (Youden´s Index)	Fertile		Subfertile	
			Mean	Range	Mean	Range
**PSV (CA)**	**0.80**	**16.00** **	**18.55**	17.37–19.72	**14.27**	12.30–16.24
**EDV (CA)**	**0.88**	**4.35** **	**5.73**	5.28–6.18	**3.87**	3.12–4.63
**TAMV (CA)**	**0.86**	**6.55** **	**8.83**	8.23–9.42	**5.89**	4.89–6.88
**TABF**	**0.83**	**0.56** **	**0.74**	0.66–0.81	**0.36**	0.23–0.49
**Artery diameter**	**0.81**	**0.29** *	**0.32**	0.31–0.34	**0.25**	0.22–0.27

The parameters assessed were: Peak systolic velocity (PSV); End diastolic velocity (EDV); Time average maximum velocity (TAMV); Total arterial blood flow (TABF) and Arterial diameter. Cut-off values were also established to

* p < 0.05;

** p < 0.01.

Interestingly, according to the PCA, fertile stallions are characterized as presenting higher values of these Doppler velocities and TABF. It means that those stallions with values of Doppler velocities and a TABF higher than cut off values will be considered fertile.

## Discussion

The breeding soundness evaluation in stallions is evermore sought after in clinical situations. This assessment includes a B-mode ultrasound examination and a basic spermiogram with a longevity test of sperm motility [7]. This imaging modality allows calculation of the testicular volume and prediction of sperm production capacity (DSOe). Stallions with chronic subfertility are characterized by a reduction in testicular volume and sperm production (oligospermic). In fact, in this study, significant differences were observed in TTV, DSOa and DSOe between fertile and sub-fertile groups (*p ≤ 0*.*05*). Nevertheless, the main problems with these parameters (TTV and DSO) are that they are unspecific and late indicators of subfertility. Normally, when DSO is affected, it is too late to implement an effective treatment [2, 6]. On the other hand, some cases of idiopathic testicular degeneration may not present any appreciable changes during a reproductive examination and only a gradual decline in sperm quality is observed. Other diagnostic techniques such as measurement of hormonal levels in plasma have been developed to try to identify stallions with testicular dysfunction [35]. This condition is frequently associated with elevated plasma FSH and LH concentrations and lower plasma estradiol concentrations. However, the measurement of these hormones is not a good predictor of the early signs of testicular dysfunction [36].

Doppler ultrasonography could be an alternative to invasive procedures such as assays to determine plasma concentrations of hormones or fine needle aspiration. This imaging modality has improved the diagnosis of testicular disorders. The blood flow of the testis is characterized by high vascular resistance that eventually triggers a low intra-testicular capillary pressure. This low pressure is responsible for a low oxygen tension in the seminiferous tubules. This low concentration is necessary for spermatogenesis [37] but it also makes the testes very susceptible to ischemic damage when any vascular disturbances reduce blood flow. For this reason, early identification of any change in testicular vascular perfusion is critical for a correct diagnosis of various testicular pathologies and promptly implementing an appropriate treatment [2].

One of the aims of this study was to assess the blood flow in fertile and subfertile stallions and to ascertain if there were differences. Total testicular perfusion was assessed by TABF and TABF rate parameters. The stallions with fertility problems in this study showed a lower vascular perfusion *(p ≤ 0*.*05)* than normal stallions. The TABF and TABF rate parameters are frequently used in human andrology as an early indicator of different pathologies such as varicocele [38]. These parameters are more sensitive than other similar velocities or Doppler indices in order to detect small changes in testicular perfusion. In this study, for the first time, we described a clear decrease in the testicular blood flow of the subfertile stallions, with a significant reduction in the diameter of the capsular artery (0.32mm vs 0.25mm). Accuracy of the parameters, to differentiate normal from sub fertile stallions was evaluated by the area under the ROC curve (AUC). Medical decision making frequently uses ROC graphs [39]. TABF and arterial diameter presented AUC values of 0.83 and 0.81 respectively. According to this statistical method, these parameters are considered to be “good indicators” for differentiating fertile stallions from those with fertility problems. Moreover, using a Youden´s Index, we have determined cut off values for both parameters. Stallions with lower values for TABF (< 0.56) and lower values for artery diameters (< 0.29) are considered subfertile (low sperm production and quality).

Conversely, Doppler ultrasound also provides several parameters that can be used as indicators of testicular efficiency since significant correlations between them and parameters of sperm production have been determined in several species [9, 12, 13]. In fact, high values of RI (RI > 0.6) measured in the intratesticular artery are associated with low sperm production in men [13]. These arteries are the vessels more frequently used to determine Doppler parameters in andrology. However, this is the first report in stallions that quantifies the blood flow parameters in the intratesticular arteries. This location of the artery presented high correlations among two Doppler velocities (EDV and TAMV) and DSOa (r_EDV_: 0.685; r_TAMV_: 0.721; *p ≤ 0*.*05*). Nevertheless, the best vessel to identify stallions with a chronic subfertility and low sperm production was the capsular artery. Once again, areas under the ROC curve over 0.8 were obtained for the parameters PSV, EDV and TAMV in the capsular artery and subsequently cut off values to identify subfertile from fertile stallions were calculated. PSV and RI have also been used in human andrology for distinguishing various causes of dyspermia [40] and in particular PSV, clearly differentiated obstructive from non-obstructive azoospermia [41]. In this study, normal stallions presented higher values of all Doppler velocities (PSV, EDV and TAMV). Additionally, according to the PCA, we also established that fertile stallions are characterized as presenting higher values of these velocities and a higher vascular perfusion (TABF and TABF rate).

At present, one of the problems in equine reproductive medicine is the fact that no objective criteria exist to assess testicular viability apart from biopsy and seminal analysis [23, 42]. The most common clinical signs presented for testicular degeneration include small, soft testes and poor semen quality with presence of immature spermatogenic cells [6]. Currently, new advances in multi-parametric flow cytometry allow simultaneous evaluation of multiple sperm compartments and functions in a large number of sperm [16, 17]. This possibility will improve sperm assessment over a short time and will establish better correlations with fertility. However, the main problem of this technique is the high cost of the equipment, and for this reason, flow cytometry is yet not widely used in routine clinical andrology. The application of other more economical and faster diagnostic tools such as Doppler ultrasound could be a good alternative for clinical practitioners. Consequently, it was also the aim of this research to study the relationship between Doppler parameters of the testicular artery and those of sperm quality assessed by flow cytometry. The indicators of seminal quality used in this work were membrane viability and integrity (YOPRO1-Eth), mitochondrial activity (Mitotracker) and the fragmentation index of chromatin (SCSA). All of these tests have been commonly used in equine andrology to diagnose fertility problems [7, 17, 43, 44]. All ejaculates were preserved at 5°C and assessed daily for three days. Normal stallions did not show an important decrease in viability and membrane integrity with time. However, the subfertile group did present a significant major percentage of dead sperm (Eth+) after 24 and 48h of preservation.

Mitochondrial activity is crucial for the functionality of sperm [44, 45]. These organelles control many spermatic functions and are considered the major sources of ROS and ATP. Under normal conditions, stallion spermatozoa present a high mitochondrial activity. However, any stress (oxidative, osmotic, etc.) could trigger mitochondrial dysfunction and sperm death [30]. In fact, mitochondria are also a good marker of apoptosis in equine sperm [46]. In this study, there were not significant differences in the percentage of live sperm with mitochondrial activity at T0 in both groups. However, the refrigeration process significantly affected mitochondria of subfertile stallions and increased the percentage of dead sperm over the time. All these factors, in addition to the higher percentage of DFI of these spermatozoa, could justify the low fertility of these horses.

The sperm chromatin structure assay has been used widely in several species to provide a prognostic value for fertility. Increased susceptibility of DNA to denaturation (% DFI) has been associated with reduced fertility in the equine [18, 32]. Pathological stallions in this study presented a major susceptibility to denaturation (DFI > 16%) in comparison to the fertile group at T0 *(p < 0*.*05)*. However, there was not an increase in the percentage of DFI over time in either group. In a previous study it was demonstrated that the stability of chromatin is not affected by cooling until 46h of preservation in healthy stallions [31]. However, in stallions with fertility problems a significant increase in the susceptibility to fragmentation of DNA was presented as early as 22h of preservation at 5°C. This could probably be due to the fact that the subfertile stallions used in that study initially presented a higher percentage of DFI (> 25%) than the subfertile stallions used in our study (>16%). However, the subfertile stallions in our work also tended to present an increase in susceptibility with time.

Several studies in other species have determined interesting correlations among Doppler indices and some parameters of semen quality such as membrane integrity and sperm motility [12, 13]. In equines, only a preliminary study shows important correlations among Doppler indices (PI and RI) and DSOa and total number of progressively motile morphologically normal sperm (TNPNS) [47]. However, the assessment of semen quality was subjective using traditional techniques such as eosin/nigrosine staining for viability.

In this study, all parameters were evaluated using a flow cytometer. To the best of our knowledge, this is the first time that correlations between Doppler parameters of the testicular artery and those of sperm quality assessed by flow cytometry have been found. The Doppler indices measured in the supratesticular artery presented a positive correlation with the subpopulation of dead sperm and those without mitochondrial activity. Doppler parameters obtained from the capsular artery were more closely correlated with sperm quality. In this location PI and RI showed a negative correlation with the percentage of live sperm, and those with a high mitochondrial activity after 24 and 48h of preservation at 5°C and a positive one with dead sperm. Thus, we could conclude that major vascular resistance may affect the tolerance of subfertile stallions to cooling. In human medicine, high values of Doppler indices are associated with ischemic or degenerative processes [8, 48]. Thus, we hypothesise that any vascular insult could trigger an ischemic process in testicular tissue. This ischemia would produce oxidative stress leading to higher percentages of damaged spermatozoa in the ejaculate more susceptible to apoptosis. These germ cells would be less tolerant of a process like refrigeration and would die faster than others when they are preserved at 5°C.

Conversely, we also determined positive correlations between RI parameters and percentage of sperm with fragmented DNA at 48h (r: 0.723, *p< 0*.*05*). Both parameters presented higher values in subfertile stallions. Once again, using the PCA we determined that stallions with poor semen quality tend to present higher values of PI and RI than normal stallions.

The Doppler velocities also presented important correlations with viability, mitochondrial activity and DNA fragmentation. EDV and TAMV were the parameters more closely correlated with quality of ejaculates at the three locations of the artery. EDV was negatively correlated with dead sperm at T0, T24 and T48 and positively correlated with intact sperm and sperm with active mitochondria at T0. Moreover, this parameter was negatively correlated with a percentage of DFI at T0, T24 and T48. These results coincide with the PCA result. Doppler velocities are the parameters, which best characterised fertile stallions.

## Conclusion

Stallions with testicular dysfunction presented a lower vascular perfusion than fertile stallions and higher Doppler index values. The better Doppler parameters to distinguish stallions with a chronic testicular dysfunction from normal stallions were: Doppler velocities (PSV, EDV and TAMV), the diameter of the capsular artery and TABF parameters (tissue perfusion parameters). Cut off values were also established in this study.

Spectral Doppler ultrasound is a good predictive tool of sperm quality in stallions since strong correlations were determined with markers of sperm quality measured by flow cytometry. Doppler ultrasonography could be a good option for clinical practitioners for the diagnosis of stallions with testicular dysfunction and could be an alternative to invasive procedures traditionally used for diagnosis of sub-fertility disorders

This study provides a firm basis for the introduction of Doppler ultrasound into stallion breeding soundness evaluations and indicates that it should be performed in all stallions with pathologies and sperm analysis abnormalities. Valuable stallions should be monitored regularly to try and identify subtle changes in blood flow over time.

## Supporting information

S1 Dataset This is the table with raw dataset of Doppler parameters. (PDF)

S2 Dataset This is the table with raw dataset of flow cytometry parameters. (PDF)

S1 File Dataset and Summary statistic of Fig 3. Dataset and Summary statistic of Table 4. Dataset and Summary statistic of Fig 4. Dataset and Summary statistic of Table 5. Dataset and Summary statistic of Fig 5. Dataset and Summary statistic of Figs 6, 7 and 8. Dataset and Summary statistic of Table 8.
(XLSX)
