# Supplementary material for: Pulse Doppler ultrasound as a tool for the diagnosis of chronic testicular dysfunction in stallions
Source: PLoS One. 2017 May 30;12(5):e0175878. doi: 10.1371/journal.pone.0175878 (PMC5448730; doi:10.1371/journal.pone.0175878)
Supplement: S1 Dataset — (PDF) [file pone.0175878.s001.pdf]

Nº stallion	Fertile/subfer	Age	Testis	IP/CT	IR/CT	PVS/CT	VDF/CT	TAMV/CT	IP/PT	IR/PT	PVS/PT
1	1	7	TI	2,42	0,82	20,50	3,60	7,00	1,28	0,67	18,40
1	1	7	TI	2,37	0,82	20,50	3,60	7,10	1,53	0,72	18,10
1	1	7	TI	2,21	0,82	20,10	3,60	7,50	1,51	0,71	18,10
1	1	7	TD	2,08	0,72	29,10	8,00	10,10	1,05	0,58	22,60
1	1	7	TD	2,31	0,76	30,30	7,40	9,90	1,10	0,58	23,70
1	1	7	TD	2,03	0,76	30,30	7,40	11,30	1,06	0,60	21,10
2	1	17	TI	2,87	0,87	29,50	3,80	8,90	1,15	0,61	20,60
2	1	17	TI	2,81	0,88	30,90	3,80	9,70	1,29	0,64	20,60
2	1	17	TI	2,99	0,88	31,90	3,80	9,40	1,55	0,66	19,50
2	1	17	TD	2,59	0,79	24,70	5,20	7,60	1,96	0,78	19,70
2	1	17	TD	2,80	0,86	25,50	3,50	7,90	1,86	0,74	19,10
2	1	17	TD	2,66	0,80	23,30	4,80	7,00	1,84	0,74	19,70
3	1	9	TI	1,47	0,72	19,20	5,40	9,40	1,54	0,68	12,90
3	1	9	TI	1,62	0,73	18,30	5,00	8,20	1,24	0,64	12,50
3	1	9	TI	1,59	0,73	16,70	4,60	7,60	1,43	0,72	24,60
3	1	9	TD	1,94	0,78	19,90	4,40	8,00	1,07	0,65	20,80
3	1	9	TD	2,00	0,75	19,40	4,90	7,30	1,22	0,66	15,50
3	1	9	TD	2,40	0,84	20,90	3,40	7,30	1,28	0,68	16,30
4	1	7	TI	1,75	0,74	28,70	7,50	12,10	1,05	0,62	11,70
4	1	7	TI	1,93	0,76	28,70	6,90	11,30	1,03	0,61	13,00
4	1	7	TI	1,90	0,76	26,20	6,20	10,50	1,06	0,62	12,30
4	1	7	TD	1,60	0,73	15,50	4,10	7,10	0,82	0,52	16,10
4	1	7	TD	1,52	0,74	20,40	5,30	9,90	0,87	0,56	13,00
4	1	7	TD	1,64	0,73	21,90	5,80	9,80	0,98	0,57	12,60
5	1	6	TI	2,62	0,80	27,80	5,40	8,50	1,08	0,63	16,50
5	1	6	TI	2,49	0,84	26,70	4,40	9,00	1,28	0,70	14,40
5	1	6	TI	2,36	0,80	21,80	4,40	7,40	1,27	0,63	17,50
5	1	6	TD	1,91	0,78	19,40	4,20	8,00	1,30	0,68	12,60
5	1	6	TD	1,88	0,77	20,20	4,60	8,30	1,37	0,63	15,50
5	1	6	TD	1,91	0,74	20,40	5,30	7,90	1,29	0,68	16,90
6	1	18	TI	2,42	0,81	28,30	5,40	9,40	1,53	0,69	17,20
6	1	18	TI	2,82	0,83	29,30	4,90	8,70	1,79	0,70	16,80
6	1	18	TI	2,93	0,84	26,60	4,30	7,60	1,80	0,71	18,70
6	1	18	TD	2,59	0,82	27,20	4,90	8,60	1,71	0,71	24,70
6	1	18	TD	2,07	0,76	26,60	6,50	9,70	1,72	0,69	25,40
6	1	18	TD	2,43	0,80	27,70	5,40	9,20	1,91	0,78	26,90
7	1	18	TI	2,75	0,87	40,50	5,20	12,80	2,39	0,81	20,40
7	1	18	TI	2,65	0,88	38,20	4,50	12,70	1,95	0,78	19,90
7	1	18	TI	2,92	0,88	45,00	5,20	13,60	2,51	0,85	18,90
7	1	18	TD	2,30	0,84	15,80	2,50	5,80	1,98	0,80	24,30
7	1	18	TD	2,67	0,85	17,10	2,50	5,50	1,98	0,82	23,80
7	1	18	TD	2,72	0,88	17,50	2,10	5,70	2,11	0,85	26,20
8	2	15	TD	1,58	0,71	35,90	10,50	16,10	1,15	0,67	12,70
8	2	15	TD	1,68	0,71	22,90	6,50	9,70	1,03	0,63	13,20
8	2	15	TD	1,51	0,71	24,30	7,00	11,40	1,15	0,63	19,30
9	2	14	TI	4,13	0,91	40,00	3,70	8,80	3,20	0,82	17,50
9	2	14	TI	4,45	0,91	35,60	3,10	7,30	3,50	0,86	17,00

9	2	14	TI	2,93	0,77	20,90	4,80	5,50	2,27	0,70	12,00
9	2	14	TD	2,82	0,75	21,00	5,20	5,60	3,06	0,74	12,80
9	2	14	TD	4,28	0,88	20,80	2,40	4,30	2,88	0,77	12,40
9	2	14	TD	3,98	0,86	20,80	2,90	4,50	2,18	0,76	12,60
10	2	14	TI	2,65	0,88	23,90	2,90	7,90	1,49	0,77	15,10
10	2	14	TI	2,62	0,84	26,20	4,10	8,50	1,91	0,77	15,90
10	2	14	TI	2,87	0,87	26,80	3,50	8,10	1,98	0,77	14,40
10	2	14	TD	1,57	0,71	24,20	6,90	11,00	1,43	0,68	12,30
10	2	14	TD	1,70	0,73	21,20	5,60	9,20	1,15	0,64	13,60
10	2	14	TD	1,90	0,80	23,40	4,80	9,80	1,50	0,73	13,30

VDF/PT	TAMV/PT	IP/IT	IR/IT	PVS/IT	VDF/IT	TAMV/IT	ø artery (cm)	A (cm^2)	TABF	TABF ratio
6,10	9,60	0,61	0,43	8,10	4,60	5,70	0,32	0,08	0,77	0,23
5,00	8,60	0,76	0,50	8,70	4,30	5,70	0,32	0,08	0,69	0,20
5,30	8,50	0,78	0,50	8,70	4,30	5,50	0,32	0,08	0,68	0,20
9,50	12,40	0,74	0,51	11,10	5,40	7,70	0,35	0,10	1,19	0,35
9,80	12,60	0,70	0,50	10,90	5,40	7,80	0,35	0,10	1,21	0,35
8,40	12,00	0,85	0,51	10,60	5,20	6,40	0,35	0,10	1,15	0,34
8,10	10,80	1,04	0,57	9,40	4,10	5,10	0,31	0,08	0,82	0,33
7,40	10,30	1,20	0,67	10,30	3,40	5,80	0,31	0,08	0,78	0,32
6,60	8,30	1,14	0,65	9,70	3,40	5,50	0,31	0,08	0,63	0,25
4,30	7,90	0,71	0,48	7,00	3,60	4,70	0,31	0,08	0,60	0,24
5,00	7,60	0,77	0,55	5,30	2,40	3,70	0,31	0,08	0,57	0,23
5,20	7,90	0,81	0,53	7,70	3,60	5,10	0,31	0,08	0,60	0,24
4,20	5,60	1,01	0,63	11,70	4,30	7,40	0,36	0,10	0,57	0,14
4,50	6,40	1,07	0,64	12,00	4,30	7,20	0,36	0,10	0,65	0,16
7,00	12,30	0,95	0,61	10,20	4,00	6,50	0,36	0,10	1,25	0,31
7,30	12,60	1,10	0,65	6,30	2,20	3,70	0,35	0,10	1,21	0,30
5,30	8,30	1,08	0,65	7,10	2,50	4,30	0,35	0,10	0,80	0,19
5,30	8,60	1,07	0,68	10,40	3,30	6,60	0,35	0,10	0,83	0,20
4,50	6,90	0,52	0,44	9,90	5,60	8,30	0,24	0,05	0,31	0,07
5,00	7,70	0,63	0,45	9,60	5,20	6,80	0,24	0,05	0,35	0,08
4,70	7,10	0,65	0,48	9,00	4,60	6,60	0,24	0,05	0,32	0,07
7,80	10,10	0,62	0,48	6,60	3,50	5,10	0,23	0,04	0,42	0,09
5,80	8,30	0,59	0,45	9,50	5,20	7,40	0,23	0,04	0,34	0,07
5,40	7,30	0,70	0,50	9,80	4,90	7,00	0,23	0,04	0,30	0,07
6,10	9,50	0,77	0,54	8,20	3,70	5,80	0,32	0,08	0,76	0,19
4,30	7,90	0,74	0,53	8,40	4,00	6,00	0,32	0,08	0,64	0,16
6,50	8,70	0,73	0,52	9,50	4,60	6,70	0,32	0,08	0,70	0,18
4,10	6,60	0,82	0,54	10,20	4,70	6,70	0,35	0,10	0,63	0,16
5,70	7,20	0,67	0,49	10,10	5,20	7,30	0,35	0,10	0,69	0,17
5,40	8,80	0,86	0,58	11,70	4,90	7,90	0,35	0,10	0,85	0,21
5,30	7,70	1,20	0,69	9,30	3,00	5,50	0,38	0,113	0,87	0,20
5,00	6,60	1,31	0,69	13,00	4,00	6,90	0,38	0,113	0,75	0,17
5,30	7,40	1,27	0,68	12,70	4,00	6,80	0,38	0,113	0,84	0,19
7,30	10,20	1,05	0,64	12,00	4,30	7,30	0,35	0,096	0,98	0,23
8,00	10,10	1,10	0,67	13,00	4,30	7,80	0,35	0,096	0,97	0,22
5,80	11,00	1,17	0,72	11,10	3,10	6,80	0,35	0,096	1,06	0,24
3,90	6,90	1,20	0,65	15,00	5,30	8,10	0,33	0,09	0,59	0,13
4,40	8,00	0,86	0,56	18,10	7,90	11,80	0,33	0,09	0,68	0,15
2,90	6,40	0,87	0,58	16,80	7,10	11,20	0,33	0,09	0,55	0,12
4,90	9,80	1,01	0,59	8,50	3,50	4,90	0,31	0,08	0,74	0,17
4,40	9,80	1,00	0,59	7,90	3,20	4,70	0,31	0,08	0,74	0,17
3,90	10,60	1,04	0,64	8,20	2,90	5,10	0,31	0,08	0,80	0,18
4,20	7,30	0,65	0,49	8,60	4,40	6,40	0,34	0,09	0,66	0,36
4,90	8,00	0,56	0,46	8,60	4,60	7,10	0,34	0,09	0,73	0,39
7,20	10,50	0,76	0,58	8,20	3,50	6,20	0,34	0,09	0,95	0,51
3,10	4,50	1,07	0,65	10,10	3,60	6,10	0,16	0,02	0,09	0,06
2,30	4,20	1,15	0,64	9,00	3,30	5,00	0,16	0,02	0,08	0,06

3,60	3,70	1,12	0,64	9,80	3,60	5,60	0,16	0,02	0,07	0,05
3,30	3,10	1,04	0,66	9,70	3,30	6,10	0,16	0,02	0,06	0,04
2,90	3,30	1,16	0,72	8,40	2,30	5,20	0,16	0,02	0,07	0,05
3,10	4,40	1,25	0,74	7,70	2,00	4,60	0,16	0,02	0,09	0,06
4,30	7,30	0,65	0,46	7,60	4,10	5,30	0,31	0,08	0,55	0,16
3,60	6,40	0,64	0,50	7,40	3,70	5,70	0,31	0,08	0,48	0,14
3,20	5,60	0,62	0,46	6,40	3,40	4,70	0,31	0,08	0,42	0,13
3,90	5,90	0,64	0,43	5,70	3,20	3,90	0,27	0,06	0,34	0,10
4,80	7,60	0,78	0,54	20,30	9,30	14,10	0,27	0,06	0,44	0,13
3,60	6,50	0,68	0,51	20,70	10,10	15,50	0,27	0,06	0,37	0,11
