# Supplementary material for: Pulse Doppler ultrasound as a tool for the diagnosis of chronic testicular dysfunction in stallions
Source: PLoS One. 2017 May 30;12(5):e0175878. doi: 10.1371/journal.pone.0175878 (PMC5448730; doi:10.1371/journal.pone.0175878)
Supplement: S2 Dataset — (PDF) [file pone.0175878.s002.pdf]

		T0				T24				T48			
**Stallion**	**Ejaculate**	**alive**	**YoPro+**	**o-pro/ Eth**	**Eth +**	**alive**	**YoPro +**	**Yo-pro/ Eth +**	**Eth +**	**Alive**	**YoPro +**	**-pro/ Eth**	**Eth +**
1	1	67,21	14,85	17,92	0,01	73,51	1,61	1,71	23,16	69,98	7,07	21,57	1,38
1	2	85,53	0,99	11,40	2,08	80,31	9,42	9,70	0,57	81,00	2,44	9,69	6,86
2	1	74,15	10,13	15,68	0,04	70,13	2,22	13,84	13,82	68,47	2,99	26,17	2,36
2	2	80,55	1,19	14,97	3,29	70,56	6,09	2,93	20,42	71,93	0,00	0,00	28,07
3	1	85,92	0,46	5,59	8,02	73,28	2,93	7,70	16,09	70,05	0,80	4,11	25,04
3	2	72,54	0,83	12,90	13,73	69,43	0,02	0,05	30,49	66,03	0,01	0,02	33,94
4	1	89,50	0,16	0,26	10,08	83,87	2,91	8,88	4,34	84,05	0,45	1,51	13,99
4	2	89,21	0,12	0,46	10,21	82,65	0,01	0,06	17,28	81,36	0,01	0,02	18,61
5	1	73,97	6,48	19,52	0,03	72,52	2,24	4,25	21,00	68,52	6,11	25,02	0,35
5	2	78,60	6,73	13,52	1,16	72,92	2,85	8,52	15,71	72,47	1,38	5,28	20,87
6	1	85,74	2,18	12,07	0,02	78,07	2,74	2,08	17,12	72,34	4,51	22,65	0,50
6	2	68,32	2,48	22,23	6,96	26,48	24,91	10,75	37,86	6,55	5,28	16,22	71,95
7	1	66,17	0,46	2,99	30,38	62,83	1,59	15,44	20,15	58,20	2,09	8,29	31,43
7	2	72,80	0,78	12,50	13,92	70,90	0,46	1,60	27,03	68,07	0,06	0,39	31,48
8	1	69,88	3,42	13,13	13,57	4,74	63,20	31,66	0,40	12,53	55,31	17,14	15,02
8	2	81,62	0,66	1,45	16,27	48,28	1,66	3,00	47,06	42,07	0,69	1,95	55,29
9	1	48,01	1,87	25,33	24,79	17,69	0,05	0,47	81,80	6,94	0,14	2,07	90,85
9	2	56,29	0,06	0,22	43,43	35,66	0,01	0,03	64,31	23,57	0,73	2,50	73,20
10	1	58,95	2,65	19,23	19,18	11,22	31,63	16,07	41,10	9,74	27,73	9,61	52,94
10	2	68,96	0,36	0,84	29,85	41,97	0,84	1,52	55,69	32,82	0,71	2,23	64,25

		T0				T24				T48			
**Stallion**	**Ejaculate**	**Mit-/Eth-**	**Mit+**	**(Mit+/Eth+)**	**Eth+**	**Mit-/Eth**	**Mit+**	**Mit+/Eth+**	**Eth+**	**Mit-/Eth**	**Mit+**	**Mit+/Eth-**	**Eth+**
1	1	12,65	71,04	0,60	15,71	6,47	69,86	4,35	19,32	13,37	68,95	1,04	16,64
1	2	3,37	84,87	1,53	10,24	15,09	78,72	0,57	5,62	6,16	78,79	1,10	13,95
2	1	1,69	82,86	0,94	14,51	4,47	68,46	4,15	22,92	5,60	66,17	15,27	12,96
2	2	0,20	81,58	17,55	0,67	6,05	70,66	22,20	1,09	4,09	67,88	27,38	0,64
3	1	0,99	85,71	4,99	8,31	6,43	70,55	6,59	16,42	5,06	66,58	2,29	26,07
3	2	0,44	73,01	24,50	2,06	3,55	65,97	24,38	6,11	6,17	60,11	27,30	6,43
4	1	1,09	88,77	1,26	8,88	4,91	82,50	0,55	12,04	2,69	82,54	1,55	13,22
4	2	1,59	87,82	4,15	6,44	2,09	80,67	11,52	5,72	5,04	76,62	5,12	13,23
5	1	11,14	71,67	0,51	16,68	1,11	74,31	20,90	3,68	8,89	68,71	5,67	16,74
5	2	1,93	83,52	10,83	3,72	5,34	70,66	11,68	12,31	7,13	67,18	10,05	15,64
6	1	1,77	86,67	1,26	10,30	1,17	80,19	15,86	2,79	5,92	71,83	11,03	11,23
6	2	0,77	70,12	28,43	0,68	20,70	31,28	40,60	7,42	0,19	12,14	87,26	0,41
7	1	0,98	65,88	8,29	24,85	3,14	61,78	8,47	26,61	4,15	57,02	7,90	30,93
7	2	0,77	72,92	16,42	9,89	2,57	68,84	16,75	11,84	3,74	64,50	16,05	15,71
8	1	4,47	73,99	12,77	8,77	0,85	76,07	19,50	3,58	11,21	68,89	18,68	1,22
8	2	0,22	82,18	15,06	2,54	0,05	51,53	46,45	1,98	0,50	46,14	50,66	2,70
9	1	0,93	49,10	34,53	15,44	0,50	17,65	64,51	17,34	0,36	7,03	76,00	16,60
9	2	1,85	54,78	23,61	19,76	1,93	35,16	39,36	23,56	2,06	24,35	46,32	27,27
10	1	2,70	61,55	23,65	12,11	0,68	46,86	42,01	10,46	5,79	37,96	47,34	8,91
10	2	1,04	68,48	19,34	11,15	0,99	43,35	42,91	12,77	1,28	35,25	48,49	14,99

Stallion	ertile/subfer	Ejaculate	SCSA/T0	SCSA/T24	SCSA/T48
1	1	1	4,19	7,71	8,35
1	1	2	6,36	6,18	6,32
2	1	1	5,13	7,31	7,51
2	1	2	4,81	5,89	5,64
3	1	1	8,80	5,78	8,21
3	1	2	5,44	6,69	7,42
4	1	1	6,90	5,02	5,12
4	1	2	5,99	7,04	5,46
5	1	1	8,33	12,13	7,58
5	1	2	7,28	8,38	9,49
6	1	1	9,05	9,65	9,64
6	1	2	9,12	9,73	9,67
7	1	1	4,74	11,00	11,06
7	1	2	10,49	13,84	17,78
8	2	1	12,73	12,92	14,25
8	2	2	14,36	15,93	12,39
9	2	1	20,88	23,07	22,38
9	2	2	12,58	19,31	23,50
10	2	1	16,81	18,00	18,32
10	2	2	13,47	17,62	17,95
